# Sleep EEG characteristics associated with total sleep time misperception in young adults: an exploratory study

**DOI:** 10.1186/s12993-022-00188-2

**Published:** 2022-01-24

**Authors:** Biyun Xu, Qinghao Cai, Runru Mai, Hailong Liang, Jiayu Huang, Zhimin Yang

**Affiliations:** 1 Department of Fangcun Sleep-Disorder, the Second Clinical College of Guangzhou University of Chinese Medicine (Guangdong Provincial Hopsital of Chinese Medicine), Guangzhou, 510120 China; 2 Applicants for Doctoral Degree with an Equivalent Educational Level in Guangzhou University of Chinese Medicine, Guangzhou, 510006 China; 3 111 Dade Road, Yuexiu District, Guangzhou, 510120 China

**Keywords:** Young adult, Misperception, EEG, Power spectral analysis, Cortical activation

## Abstract

**Background:**

Power spectral analysis (PSA) is one of the most commonly-used EEG markers of cortical hyperarousal, and can help to understand subjective–objective sleep discrepancy (SOD). Age is associated with decreased sleep EEG activity; however, the PSA of young adults is currently limited. Thus, this study aimed to examine the correlation of spectral EEG power with total sleep time (TST) misperception in young patients.

**Methods:**

Forty-seven young adults were recruited and underwent a polysomnography recording in a sleep laboratory. Clinical records and self-report questionnaires of all patients were collected, and were used to categorize patients into a good sleeper (GS) group (n = 10), insomnia with a low mismatch group (IWLM, n = 19) or participant with a high mismatch group (IWHM, n = 18). PSA was applied to the first 6 h of sleep.

**Results:**

IWHM patients exhibited a higher absolute power and relative beta/delta ratio in the frontal region compared to the GS group. No significant difference was observed between the IWLM and GS groups. No significant difference in the above parameters was observed between the IWHM and IWLM groups. Moreover, The SOD of TST was positively correlated with frontal absolute power and the relative beta/delta ratio (r = 0.363, *P* = 0.012; r = 0.363, *P* = 0.012), and absolute beta EEG spectral power (r = 0.313, *P* = 0.032) as well as the number of arousals.

**Conclusions:**

Increased frontal beta/delta ratio EEG power was found in young patients with a high mismatch but not in those with a low mismatch, compared with good sleepers. This suggests that there exists increased cortical activity in IWHM patients. In addition, the frontal beta/delta ratio and the number of arousals was positively correlated with the SOD of TST.

## Introduction

Insomnia is a common disease in modern society with a prevalence rate ranging from 12 to 20% [1]. Patients with insomnia have a higher risk of mental illness [2] and physical diseases [3, 4], which may lead to higher healthcare and medical costs, higher chances of absenteeism, traffic accidents, falling and a poor quality-of-life [4–7].

Subjective–objective sleep discrepancy (SOD), also called sleep misperception, refers to the underestimation of total sleep time (TST) and overestimation of sleep onset latency (SOL). The SOD of TST is obtained by subtracting self-reported TST (sTST) values from objective TST (oTST) values detected by polysomnography (PSG) [8]. SOD is very common in insomnia patients [9, 10] and is extremely common in paradoxical insomnia which was listed in the International Classification of Sleep Disorders-2nd Edition (ICSD-2), but cancelled in ICSD-3 mainly due to a lack of consensus on its precise definition. Although it was cancelled, the symptom is worth studying [11] since it is an important aspect for understanding the mechanism of insomnia.

Several studies have attempted to explain the possible mechanisms of SOD in insomnias and have accumulated 13 possible mechanisms thereof that are supported by good-quality evidence [9]. One of these potential mechanisms is related to cortical hyperarousal. Power spectral analysis (PSA) is one of the most commonly-used EEG markers of cortical hyperarousal [12]. PSA can show whether information processing is in an enhanced state, which may cause the misperception of sleeping state [13]. The power of each waveform is defined as the area under the waveform, where a high amplitude represents high power [9].

Previous studies have shown that patients with primary insomnia (PI) have more high-frequency EEG activity, especially beta-band activity, during non-rapid eye movement (NREM) sleep [14–18] than healthy controls. This finding is also consistent with the hyperarousal theory of insomnia. However, there is still no consensus on other power bands during NREM sleep. For example, a meta-analysis including 532 patients with insomnia disorder (ID) and 445 good sleepers performed by Zhao et al. [18] found that patients with ID exhibited increased theta, alpha, and sigma power during NREM sleep. Riedner et al. [17] found that ID patients exhibited no difference in slow wave (specifically < 5 Hz) and sigma (spindle) frequencies (specifically 11–16 Hz) compared with GS. This difference could be explained by a number of reasons, one of which is the difference in various subtypes of insomnia. Many of the diseases studied fall under the umbrella term of PI, which is a broad term that includes paradoxical insomnia (ParI) and psychophysiological insomnia (PsyI), as well as idiopathic insomnia. However, some subtypes, for example [9] individuals with and without sleep misperception, are not considered in most studies. Therefore, it has been suggested that future studies on EEG spectral features should patients from different insomnia subtypes. To our knowledge, only several studies aimed to investigate SOD to understand the underlying mechanism of insomnia. Krystal et al. [19], St-Jean et al. [20] and Lecci et al. [21] categorized their patients according to SOD, but there was no consistency in the spectral power analyses in these studies and only elderly patients (40–80 years old vs. 40.21 + 9.38 vs 40–85 years old) were included. Krystal et al. [19] reported that lower delta and greater alpha, sigma and beta NREM EEG activity was found in patients with subjective insomnia but not in those with objective insomnia, compared with normal subjects. St-Jean et al. [20] reported that patients with ParI exhibited higher absolute delta activity at the standardized F3, C3, and P3 electrodes compared to those with PsyI.

It has also shown that there is a relationship between age and s decrease of sleep EEG activity [22, 23]. Compared to young individuals, elderly patients exhibit less N3 and spindle activity during non-rapid eye movement (NREM) sleep, and a smaller proportion of rapid eye movement (REM) sleep [24]. It has also been shown that absolute power in the delta, theta and sigma bands decline with age for both females and males [23]. Importantly, previous research has failed to combine age data with insomnia subtype information to study the PSA of SOD.

In this study, we aimed to compare the PSA of three groups of young patients: (1) patients who overestimated their total sleep time by at least 2 h; (2) patients who correctly estimated their sleep; (3): good sleepers (GS). Our findings may be important to clinical and public health as well as the treatment and management of insomnia [9].

## Materials and methods

### Participants

Seventy participants aged between 18 and 40 years-old were recruited from the Guangdong Provincial Hospital of Chinese Medicine through posters from May 2016 to November 2017. All subjects were asked to complete a two-week sleep diary followed by a single all-night PSG recording in a sleep laboratory. Personal information was obtained from all subjects, including age, sex, race, place of residence, marital status, family history of insomnia and psychosis. Two self-reported questionnaires, the Pittsburgh Sleep Quality Index (PSQI) [25] and the Symptom Checklist 90 (SCL-90), were given to each participant. Subjective sleep quality was determined by self-reported TST after PSG. The subjects were asked two questions about their perceived sleep within 2 h after PSG completion: (1) “How long did you sleep last night?” and (2) “Did you sleep as usual?”. In this way, the sTST of the patient was obtained. For example, if the patient replied that they slept for 6 h during the previous night, 360 min was his/her sTST.

Subjects were categorized as GS according to the following criteria: (1) reported no difficulty in sleep according to the two-week sleep diary (i.e. sleep onset (SO) < 30 min, wake after sleep onset (WASO) < 40 min, TST between 6.0 and 8.0 h, or sleep efficiency (SE) ≥ 85%); (2) had a PSQI score < 7 [25], SE > 85% or TST > 6 h.

Participants were categorized as insomnia patients if they met the following criteria: (1) diagnosed with chronic insomnia disorder (International Classification of Sleep Disorders, 3rd edition); (2) reported at least three nights per week of sleep difficulty (i.e.SO > 30 min, WASO > 40 min, sTST < 6.0 h, or SE < 85%); (3) had a PSQI score of > 7; (4) had difficulty sleeping for more than 3 months; (5) did not have other medical, psychological, or sleeping disorders and did not take any medications that would affect sleep (e.g. sedative and hypnotic drugs, antidepressants, anti-schizophrenia drugs, etc.).

Insomnia patients were further categorized into two subgroups based on their SOD of TST. These two subgroups comprised patients with low mismatch (IWLM) and patients with high mismatch (IWHM). The SOD of TST was operationalized as the values of the differences between subjective and objective measures (i.e. sTST–oTST value) [8]. IWLM patients were those individuals who met the criteria of chronic insomnia disorder and had an SOD < 60 min in TST. IWHM were defined as patients who met the criteria of chronic insomnia disorder and had normal PSG parameters (i.e. SE > 85% and TST > 6.5 h) and SOD > 120 min in TST. In both the IWHM and IWLM subgroups, patients were excluded if they met one of the following criteria: (1) diagnosed with another Axis I disorder or any other sleeping disorder (e.g. idiopathic insomnia, sleep apnea, which was defined as an apnea–hypopnea index of more than five events per hour using PSG, or restless leg syndrome); (2) affected by other external factors that might affect insomnia (e.g. physical pain caused by medical diseases, drugs affecting sleeping structure, alcohol consumption, other treatments, etc.); (3) go to sleep later than 0:00 am or wake up before 6:00 am, or had irregular sleeping schedules.

Based on the inclusion and exclusion criteria, 47 participants were included in the study: GS group (n = 10; 5 males, 5 females), IWHM group (n = 18; 9 males, 9 females), and IWLM group (n = 19; 3 males, 16 females).

### PSQI and SCL-90

The PSQI is a questionnaire consisting of 21 items and has been commonly used to evaluate subjective sleep quality. The higher the score, the greater the severity of insomnia. A score > 7 indicates abnormal sleeping (severe difficulty in at least two areas or moderate difficulty in more than three areas).

The SCL-90 is one of the most widely used mental health scales in the field of psychiatry. It is a 90-item, self-reported symptom inventory. The score for each item is summed, yielding a total score that covers ten aspects. The higher the total score, the greater the risk of developing psychological distress [26].

### PSG recordings

In conventional PSG (Nicolet, ONE, EEG 32, USA), the international 10–20 system was used to record EEG. In this study, the grounding electrode was placed on the frontal pole midline point and the bilateral ear electrodes were used as the reference. All electrographic electrodes were placed according to the AASM 2.6 recommended guidelines. The impedance was kept below 5 kΩ for all electrodes. The surface electrodes included six EEG (two central electrodes [C3, C4], two frontal EEG electrodes [F3, F4], and two occipital EEG electrode [O1, O2)]), two electro-oculogram (E1, E2), submental electromyogram (EMG: Chin1-Chin2), electrocardiogram (ECG), and two reference electrodes (A1, A2). In addition, tibialis EMG and respiration were used to exclude periodic limb movements (a PLMSI > 15) and sleep apnea (an apnea–hypopnea index > 5), respectively. Participants were asked to sleep at their usual time (before 0:00 am) and wake up at 7:00 am. The sampling rate of EEG was 500 Hz and the filter settings were as follows: notch frequency at 60 Hz; low pass filter at 35 Hz; high pass filter at 0.3 Hz.

Sleep records were reviewed and scored by a registered PSG technician according to the revised AASM 2.5 sleeping scoring criteria [27]. The sleeping continuity parameters, including TST, SPT, SE (ratio of TST to time in bed × 100%), and SOL, and sleeping architecture parameters, including the number of awakenings, the number of arousals, arousal index, percentage of NREM stage 1 and 2, slow wave sleep (SWS) or NREM stage 3, and REM sleep of TST were analyzed.

### Spectral analysis

Normal sleep time is 6.0 to 8.0 h, and therefore we analyzed the first 6 h of the PSG recordings. The data from the central and frontal EEG electrode (averaged C3-A2 and C4-A1 channels, averaged F3-A2 and F4-A1 channels) were generated using software of Nicolet EEG band width tools.

Most of the common artifacts were due to improper click placements (such as electrode popping, ECG or pulse artifact), body movement (muscle artifact, eye movement artifact or major body movement) or environmental factors (overheated which lead to slow-frequency artifacts). We optimized the mastoid electrodes so that ECG and pulse artifacts could be minimized. Secondly, we kept impedance below 5 kΩ to avoid electrode popping. At the same time, we maintained a temperature of 20 °C in the sleep laboratory which is the standard setting to ensure that the subjects completed the test in a comfortable environment, and avoided the influence of slow-frequency artifacts from sweat. A notch filter at 50 Hz was applied to avoid power line contamination of the electrical signals. Then, we set a high frequency filter to 35 Hz to reduce most of the interference from EMG. We chose this cutoff values as the frequency of EMG activity signal is generally contained in higher frequency bands and since the AASM recommend that EMG low frequency and high frequency filter cutoffs should be at 10 Hz and 100 Hz, respectively, to capture the muscle activity. Finally, the data fragments that were displaced or cut off due to movements or that were obviously different from the background were to excluded by visual inspection (e.g. due to the excessive loss of occipital EEG electrode signal, these data were not included in this study). Therefore, artifacts in each recording were visually inspected and removed accordingly.

The beta (16–32 Hz), sigma (12–16 Hz), alpha (8–12 Hz), delta (0.5–4 Hz), and theta (4–8 Hz) band activity was extracted for PSA analysis. The values of relative spectral power were calculated by dividing the absolute power of each frequency band by the power of the total power spectrum.

### Statistical analysis

Statistical analysis was performed using the SPSS software (ver. 24.0) and with an unpaired two-tailed test of significance. A normality test and Levene’s test were used to check whether the data followed a normal distribution. A Chi-square test was used for demographic characteristics except for age. Normally distributed data with homogeneous variance were compared using a one-way ANOVA, while others were compared using a non-parametric analysis (Kruskal–wallis) with post-hoc analysis. The statistical value H represents the use of non-parametric analysis, while the statistical value F represents the use of a one-way ANOVA. In addition, we used pairwise least significant difference post-hoc tests after a one-way ANOVAs and a Bonferroni correction for multiple comparison after a Kruskal–Wallis test. Spearman’s or Pearson’s correlation analysis was used to determine the correlation between the EEG spectral power (absolute and relative) and the SOD of TST (after data normality was confirmed). A *P-*value < 0.05 was considered statistically significant.

## Results

### Baseline characteristics

There was no significant differences in age, sex, race, place of residence, marital status, family history of insomnia, or family history of psychosis among the three groups (Table 1).

**Table 1:** Demographic characteristics of all participants

Variables	GS (n = 10)	IWHM (n = 18)	IWLM (n = 19)	Statistics
Age, years	26.0 [25.0, 32.0]	30.5 [25.8, 38.2]	25.0 [23.0, 38.0]	H = 3.378, *p* = 0.185
Sex (F/M)	5/5	9/9	16/3	χ^2^ = 5.616, *p* = 0.060
Race				χ^2^ = 0.000, *p* = 1.000
Han	10	18	19	
Non-Han	0	0	0	
Place of residence				χ^2^ = 1.233, *p* = 0.540
Downtown	9	13	15	
Suburb	1	4	3	
Village	0	1	1	
Marriage				χ^2^ = 2.798, *p* = 0.247
Unmarried	7	7	10	
Married	3	10	9	
Bereavement/divorce	0	1	0	
Family history of insomnia (Y/N)	1/9	3/15	1/18	χ^2^ = 1.243, *p* = 0.537
Family history of psychosis (Y/N)	0/10	0/18	1/18	χ^2^ = 1.474, *p* = 0.479

GS: good sleeper; IWLM: insomnias with a low mismatch; IWHM: insomnias with a high mismatch

### PSQI, SCL90, and PSG characteristics

The comparisons of the PSQI score, SCL-90 score, and PSG among the three groups are shown in Table 2. The IWHM and IWLM groups showed higher PSQI and SCL-90 scores compared to the GS group. However, there was no significant difference in the PSQI or SCL-90 scores between the IWHM and IWLM groups. The PSG parameters were not significantly different among the GS, IWHM, and IWLM groups.

**Table 2:** PSQI scores, SCL90 scores, and PSG characteristics of all participants

Variable	GS (n = 10)	IWHM(n = 18)	IWLM (n = 19)	Statistics
PSQI total score	3.5 [2.0, 6.2]	13.5 [11.5,16.3]^a^	11.0 [9.0, 14.0]^a^	H = 18.882, *P* < 0.001
SCL-90 total score	109.5 [103.5, 129.3]	174.5 [155.8, 223.8]^a^	148.0 [127.5, 180.0]^a^	H = 16.037, *P* < 0.001
TST (min)	394.25 ± 45.25	415.06 ± 40.20	381.95 ± 39.85	F = 3.026, *P* = 0.059
SPT (min)	418.60 ± 47.39	459.03 ± 42.65	431.84 ± 49.77	F = 2.837, *P* = 0.069
SE, %	89.27 ± 3.92	87.84 ± 7.00	86.24 ± 8.39	F = 0.626, *P* = 0.540
SOL (min)	11.50 [7.13, 23.38]	8.25 [4.00, 12.63]	5.50 [3.00, 10.00]	H = 2.697, *P* = 0.260
%NREM stage1	5.00 [3.00, 6.25]	5.00 [4.00, 8.50]	4.00 [3.00, 7.00]	H = 1.158, *P* = 0.560
%NREM stage2	59.40 ± 8.51	61.17 ± 8.61	62.16 ± 7.63	F = 0.371, *P* = 0.692
% SWS	13.60 ± 4.79	10.06 ± 6.49	12.37 ± 4.82	F = 1.528, *P* = 0.228
%REM	21.90 ± 6.61	22.61 ± 3.27	19.31 ± 5.14	F = 2.259, *P* = 0.116
Number of awakenings	23.50 [19.50, 29.50]	24.00 [16.00, 31.00]	19.00 [13.00, 27.00]	H = 2.147, *P* = 0.342
Number of arousals	17.00 [8.25,20.50]	24.00 [12.75, 47.50]	26.00 [17.00, 47.00]	H = 3. 730, *P* = 0.155
Arousal index	2.69 [1.22, 3.21]	3.71 [1.77,7.42]	4.17 [2.28,7.55]	H = 3. 611, *P* = 0.164
Number of arousals of NREM	15.00 [8.25, 20.50]	19.50 [10.00, 46.50]	22.00 [17.00, 41.00]	H = 3.233, *P* = 0.199
Number of arousals of REM	0.00 [0.00, 3.00]	1.00 [0.00, 3.25]	1.00 [0.00, 5.00]	H = 1.339, *P* = 0.512

GS: good sleeper; IWLM: insomnias with a low mismatch; IWHM: insomnias with a high mismatch

PSQI: Pittsburgh Sleep Quality Index; TST: total sleep time; SPT: sleep period time; SE: sleep efficiency; SOL: sleep onset latency; NREM: non-rapid eye movement; SWS: slow wave sleep; REM: rapid eye movement

The statistical value H represents the use of a Kruskal–Wallis non-parametric analysis among three group using a Bonferroni correction for post-hoc analysis, while the statistical value F represents the use of a one-way ANOVA using the least significant difference test for post-hoc analysis

^a^*P* < 0.05 versus GS. There was no difference between the IWHW and IWLW groups

### Absolute EEG spectral power

**Fig. 1:**
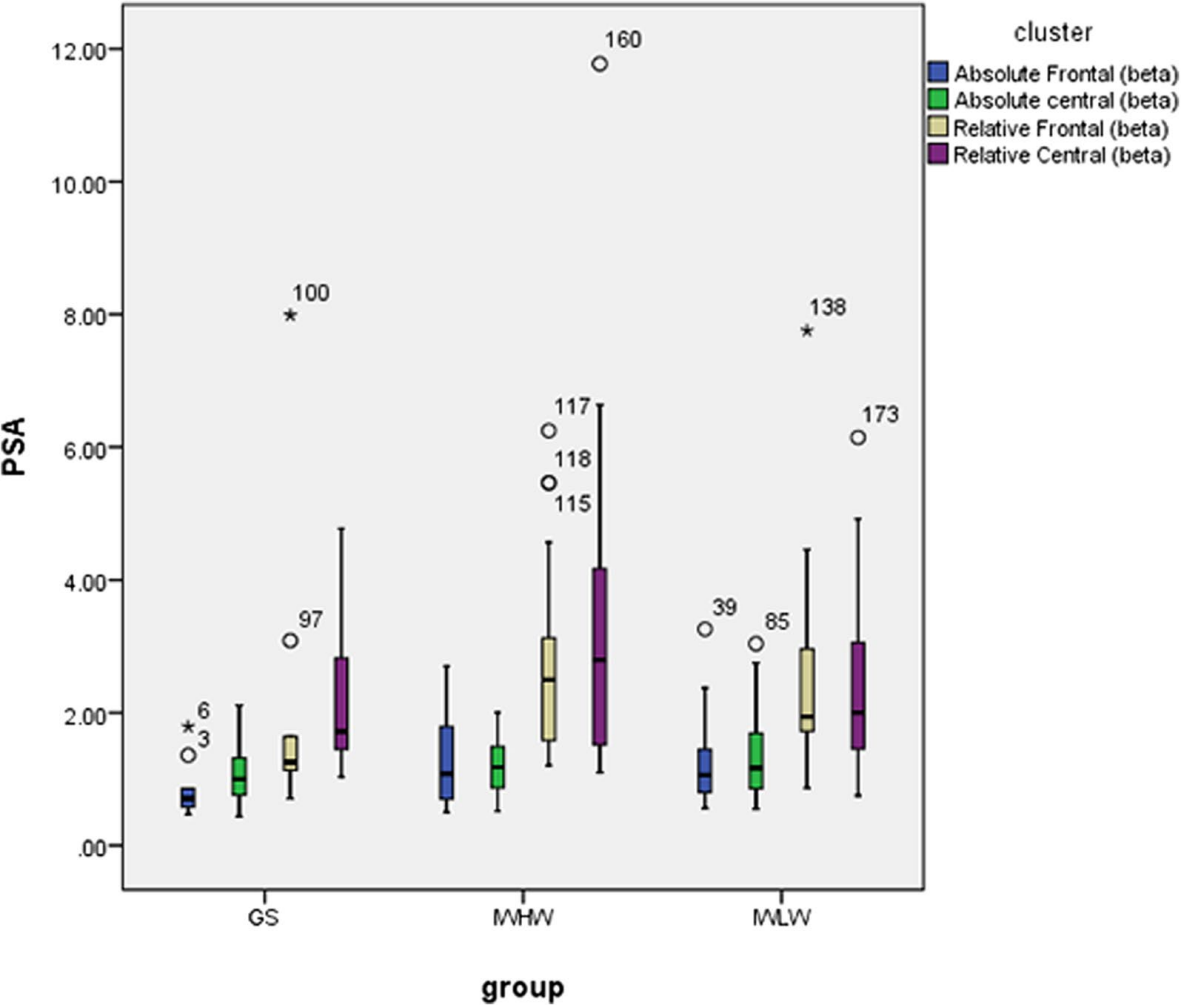
Absolute and relative beta in the frontal and central region. GS: good sleeper; IWLM: insomnias with a low mismatch; IWHM: insomnias with a high mismatch

**Fig. 2:**
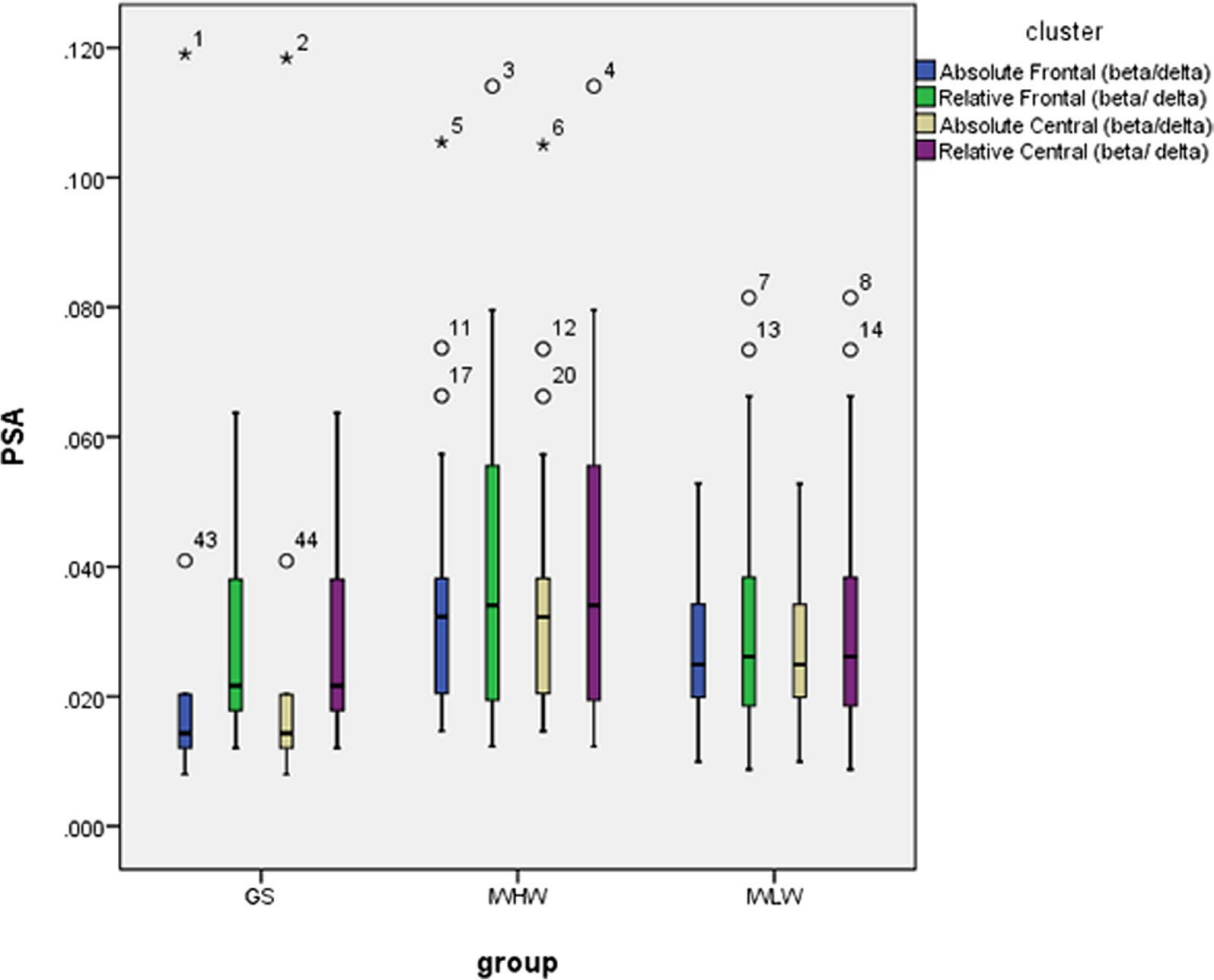
Absolute power and relative beta/delta in the frontal and central regions. GS: good sleeper; IWLM: insomnias with a low mismatch; IWHM: insomnias with a high mismatch

Post-hoc analysis (Bonferroni correction) revealed that the IWHM group exhibited a significantly higher frontal beta/delta ratio than the GS group. No significant difference was observed between the IWLM and GS groups. There was no significant difference in these parameters between the IWHM and IWLM groups (Table 3, Figs. 1, 2).

**Table 3:** Comparison of absolute EEG spectral power among the experimental groups (μν^2^)

Variable	GS (n = 10)	IWHM (n = 18)	IWLM (n = 19)	Statistics
Frontal derivation				
Delta (1–4 Hz)	46.47 ± 21.05	37.61 ± 15.30	46.65 ± 25.95	F = 0.981, *P* = 0.383
Theta (4–8 Hz	2.90 [2.29, 4.22]	3.23 [2.67, 3.99]	4.91 [2.59, 5.83]	H = 3.481, *P* = 0.175
Alpha (8–12 Hz)	1.87 [1.16, 3.37]	2.53 [1.55, 2.99]	2.78 [1.97, 3.89]	H = 2.693, *P* = 0.260
Sigma (12–16 Hz)	0.80 [0.55, 1.27]	1.09 [0.69, 1.56]	1.40 [0.88, 1.76]	H = 4.137, *P* = 0.126
Beta (16–32 Hz)	0.71 [0.57, 0.99]	1.08 [0.69, 1.80]	1.06 [0.79, 1.50]	H = 4.062, *P* = 0.131
Beta /Delta	0.01 [0.01, 0.03]	0.03 [0.02, 0.04] ^a^	0.03 [0.02, 0.04]	H = 6.904, *P* = 0.032
Beta/Theta	0.27 [0.17, 0.37]	0.34 [0.23, 0.53]	0.25 [0.18, 0.36]	H = 1.907, *P* = 0.385
Alpha/Delta	0.04 [0.03, 0.08]	0.07 [0.04, 0.09]	0.07 [0.05, 0.11]	H = 3.086, *P* = 0.214
Alpha/Theta	64.00 ± 16.43	76.60 ± 41.44	74.33 ± 30.73	F = 0.487, *P* = 0.618
Central derivation				
Delta (1–4 Hz)	37.99 ± 12.36	33.89 ± 19.01	46.48 ± 20.94	F = 2.154, *P* = 0.128
Theta (4–8 Hz	4.77 [3.37, 4.99]	4.11 [3.21, 4.63]	4.93 [2.98, 8.85]	H = 2.872, *P* = 0.238
Alpha (8–12 Hz)	2.51 [1.66, 3.24]	2.79 [1.89, 3.52]	2.70 [1.83, 4.02]	H = 0.710, *P* = 0.701
Sigma (12–16 Hz)	1.43 [1.04, 1.84]	1.66 [1.21, 2.76]	1.75 [1.37, 2.51]	H = 2.625, *P* = 0.269
Beta (16–32 Hz)	0.81 [0.66, 1.25]	1.29 [0.87, 1.62]	1.16 [0.72, 1.65]	H = 2.139, *P* = 0.343
Beta/Delta	0.02 [0.02, 0.04]	0.04 [0.02, 0.06]	0.03 [0.02, 0.04]	H = 3.087, *P* = 0.214
Beta/Theta	0.18 [015, 0.31]	0.28 [0.20, 0.43]	0.20 [0.15, 0.35]	H = 2.385, *P* = 0.303
Alpha/Delta	0.07 [0.04, 0.09]	0.08 [0.05, 0.14]	0.06 [0.05, 0.09]	H = 2.632, *P* = 0.268
Alpha/Theta	0.57 [0.47, 0.66]	0.58 [0.51, 0.92]	0.52 [0.42, 0.75]	H = 1.774, *P* = 0.412

GS: good sleeper; IWLM: insomnias with a low mismatch; IWHM: insomnias with a high mismatch. The statistical value H represents the use of a Kruskal–Wallis non-parametric analysis among three group using a Bonferroni correction for post-hoc analysis, while the statistical value F represents the use of a one-way ANOVA using the least significant difference test for post-hoc analysis

^a^*P* < 0.05 versus GS. There was no difference between the IWHW and IWLW groups

### Relative EEG spectral power

The average NREM activity for the beta/delta ratio in the frontal area was significantly different among the three groups. Post-hoc analysis (Bonferroni correction) showed that the frontal beta/delta ratio in the IWHM group was higher than that in the GS group. No significant difference was observed between the IWLM and GS groups and no significant difference in relative spectral power was observed between the IWHM and IWLM groups (Table 4, Figs. 1, 2).

**Table 4:** Comparison of relative EEG spectral power among the experimental groups

Variable	GS (n = 10)	IWHM (n = 18)	IWLM (n = 19)	Statistics
Frontal derivation				
Delta (1–4 Hz)	87.96 [79.34, 90.48]	80.74 [75.23, 83.99]	80.81 [76.26, 85.81]	H = 5.046, *P* = 0.080
Theta (4–8 Hz	5.85 [4.54, 8.54]	7.39 [6.24, 9.56]	7.20 [6.61, 10.78]	H = 3.820, *P* = 0.148
Alpha (8–12 Hz)	3.77 [2.49, 6.48]	5.08 [3.56, 6.84]	5.10 [3.80, 8.26]	H = 2.767, *P* = 0.251
Sigma (12–16 Hz)	1.31 [1.16, 2.71]	2.51 [1.75, 3.35]	2.15 [1.62, 4.32]	H = 3.498, *P* = 0.174
Beta (16–32 Hz)	1.26 [1.09, 2.00]	2.50 [1.54, 3.49]	1.94 [1.65, 2.99]	H = 5.756, *P* = 0.056
Beta /Delta	0.01 [0.01, 0.03]	0.03 [0.02,0.04]^a^	0.03 [0.02, 0.04]	H = 6.904, *P* = 0.032
Beta/Theta	0.27 [0.17, 0.37]	0.34 [0.23, 0.53]	0.25 [0.18, 0.36]	H = 1.907, *P* = 0.385
Alpha/Delta	0.04 [0.03, 0.08]	0.07 [0.04, 0.09]	0.07 [0.04, 0.11]	H = 3.183, *P* = 0.204
Alpha/Theta	64.00 ± 16.42	76.60 ± 41.44	74.32 ± 30.73	F = 0.487, *P* = 0.618
Central derivation				
Delta (1–4 Hz)	78.42 [74.84, 86.28]	74.67 [66.94, 82.17]	77.34 [74.20, 82.11]	H = 3.930, *P* = 0.140
Theta (4–8 Hz	10.04 [6.57, 10.67]	9.98 [8.57, 11.37]	9.54 [8.07, 12.22]	H = 0.281, *P* = 0.869
Alpha (8–12 Hz)	5.74 [3.51, 7.13]	5.94 [4.59, 9.21]	4.84 [4.01, 7.00]	H = 2.483, *P* = 0.289
Sigma (12–16 Hz)	3.11 [2.35, 4.39]	4.39 [3.15, 6.77]	3.29 [2.62, 4.55]	H = 5.447, *P* = 0.066
Beta (16–32 Hz)	1.72 [1.43, 3.05]	2.79 [1.51, 4.42] ^a^	2.00 [1.45, 3.38]	H = 3.121, *P* = 0.210
Beta /Delta	0.02 [0.02, 0.04]	0.04 [0.02, 0.06]	0.03 [0.02, 0.04]	H = 3.087, *P* = 0.214
Beta/Theta	0.18 [0.15, 0.31]	0.28 [0.20, 0.43]	0.20 [0.15, 0.35]	H = 2.385, *P* = 0.303
Alpha/Delta	0.07 [0.04, 0.09]	0.08 [0.05, 0.14]	0.06 [0.05, 0.09]	H = 2.632, *P* = 0.268
Alpha/Theta	0.57 [0.47, 0.66]	0.58 [0.51, 0.92]	0.52 [0.42, 0.75]	H = 1.774, *P* = 0.412

GS: good sleeper; IWLM: insomnias with a low mismatch; IWHM: insomnias with a high mismatch

The statistical value H represents the use of a Kruskal–Wallis non-parametric analysis among three group using a Bonferroni correction for post-hoc analysis, while the statistical value F represents the use of a one-way ANOVA using the least significant difference test for post-hoc analysis. ^a^*P* < 0.05 versus GS. There was no difference between the IWHW and IWLW groups

### Correlation between absolute EEG spectral power and SOD

**Fig. 3:**
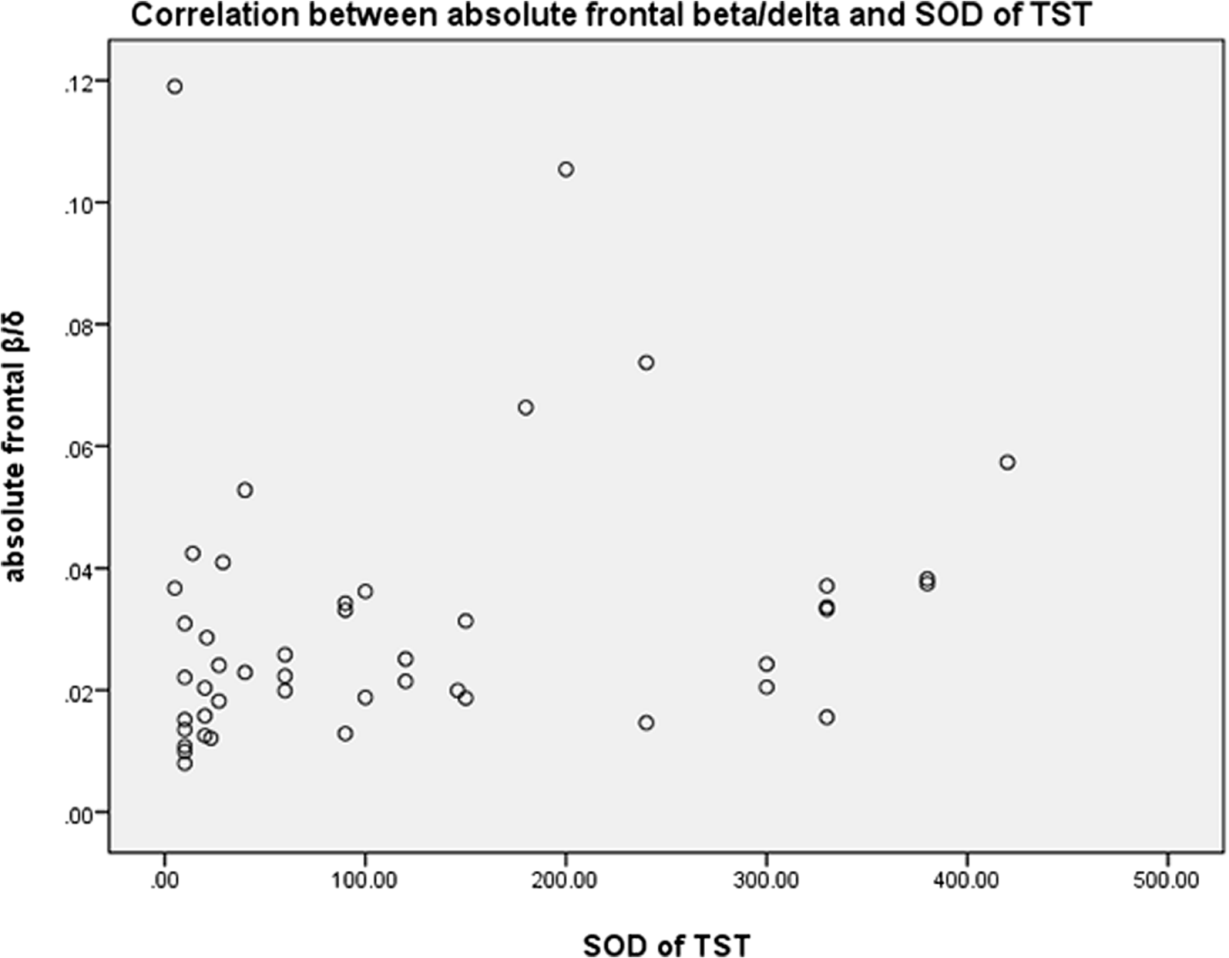
Correlation between absolute frontal beta/dela spectral power and SOD of TST. SOD: Subjective–objective sleep discrepancy; TST: total sleep time

Spearman’s correlation was performed on the SOD due to the non-normality of the data. The SOD of TST was positively correlated with absolute frontal beta/delta ratio (r = 0.363, *P* = 0.012) (Table 5, Fig. 3).

**Table 5:** Correlation between absolute EEG spectral power and SOD of TST

Variables	SOD of TST	
	r	*P*
Frontal derivation		
Delta (1–4 Hz)	− 0.202	0.173
Theta (4–8 Hz)	− 0.032	0.833
Alpha (8–12 Hz)	0.058	0.698
Sigma (12–16 Hz)	− 0.013	0.931
Beta (16–32 Hz)	0.200	0.179
Beta/Delta	0.363	0.012
Beta/Theta	0.208	0.161
Alpha/Delta	0.171	0.249
Alpha/Theta	0.182	0.221
Central derivation		
Delta (1–4 Hz)	− 0.109	0.467
Theta (4–8 Hz	− 0.063	0.673
Alpha (8–12 Hz)	0.188	0.205
Sigma(12–16 Hz)	0.156	0.295
Beta (16–32 Hz)	0.216	0.145
Beta/Delta	0.249	0.091
Beta/Theta	0.188	0.256
Alpha/Delta	0.256	0.082
Alpha/Theta	0.169	0.257

SOD: Subjective–objective sleep discrepancy; TST: total sleep time

### Correlation between relative EEG spectral power and SOD

**Fig. 4:**
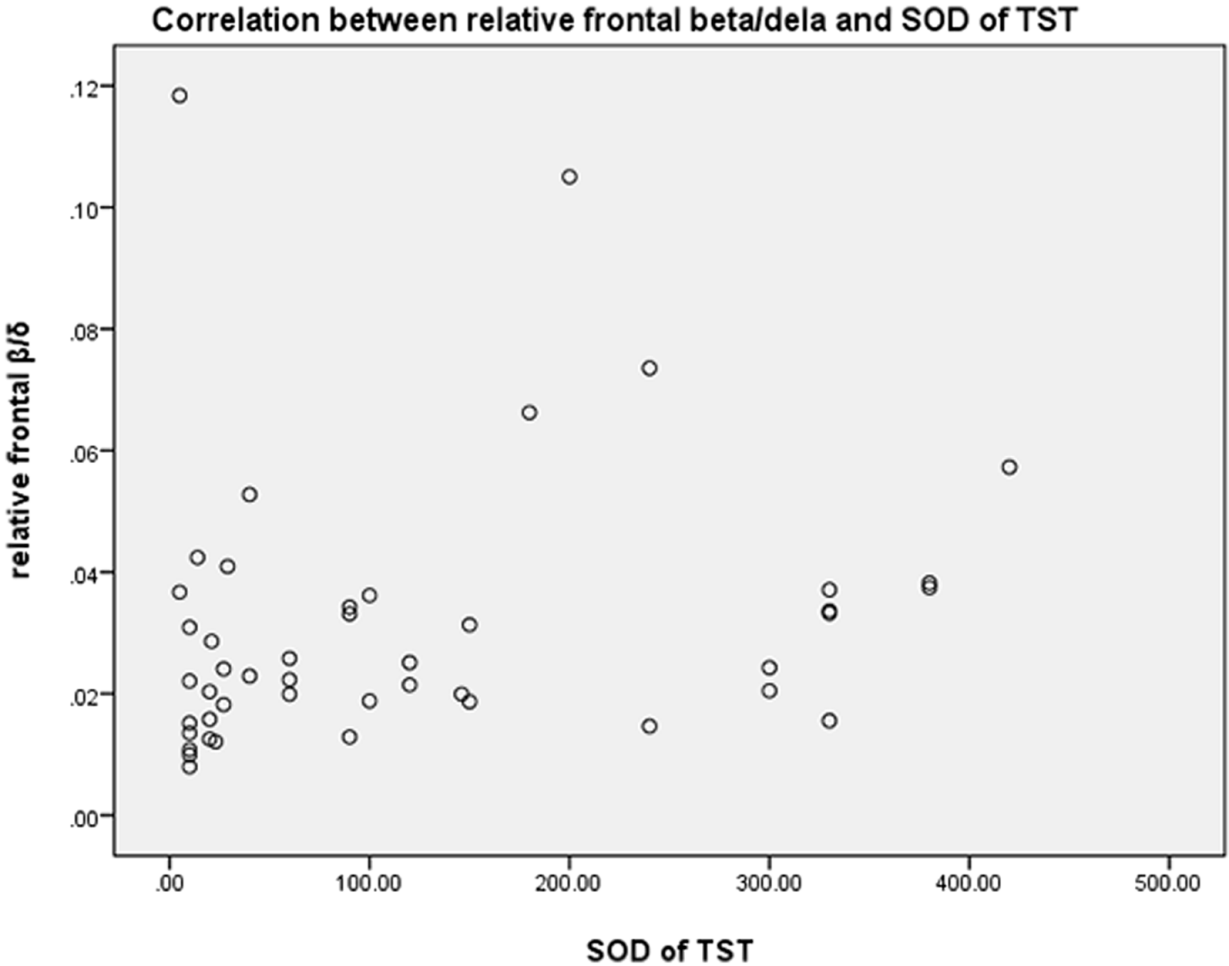
Correlation between relative frontal beta/dela spectral power and SOD of TST. SOD: Subjective–objective sleep discrepancy; TST: total sleep time

Spearman’s correlation was performed on the SOD due to the non-normality of the data. The SOD of TST was positively correlated with relative frontal beta/delta ratio (r = 0.363, *P* = 0.012) and the absolute beta EEG spectral power (r = 0.313, *P* = 0.032) (Table 6, Fig. 4).

**Table 6:** Correlation between relative EEG spectral power and SOD of TST

Variables	SOD of TST	
	r	*P*
Frontal derivation		
Delta (1–4 Hz)	− 0.248	0.092
Theta (4–8 Hz	0.127	0.397
Alpha (8–12 Hz)	0.158	0.290
Sigma (8–12 Hz)	0.124	0.405
Beta (16–32 Hz)	0.313	0.032
Beta/Delta	0.363	0.012
Beta/Theta	0.208	0.161
Alpha/Delta	0.173	0.245
Alpha/Theta	0.182	0.221
Central derivation		
Delta (1–4 Hz)	− 0.268	0.069
Theta (4–8 Hz	− 0.072	0.631
Alpha (8–12 Hz)	0.247	0.094
Sigma (8–12 Hz)	0.207	0.163
Beta (16–32 Hz)	0.232	0.116
Beta/Delta	0.249	0.091
Beta/Theta	0.1888	0.205
Alpha/Delta	0.256	0.082
Alpha/Theta	0.169	0.257

SOD: Subjective–objective sleep discrepancy; TST: total sleep time

### Correlation between number of arousals and frontal beta and SOD of TST

**Fig. 5:**
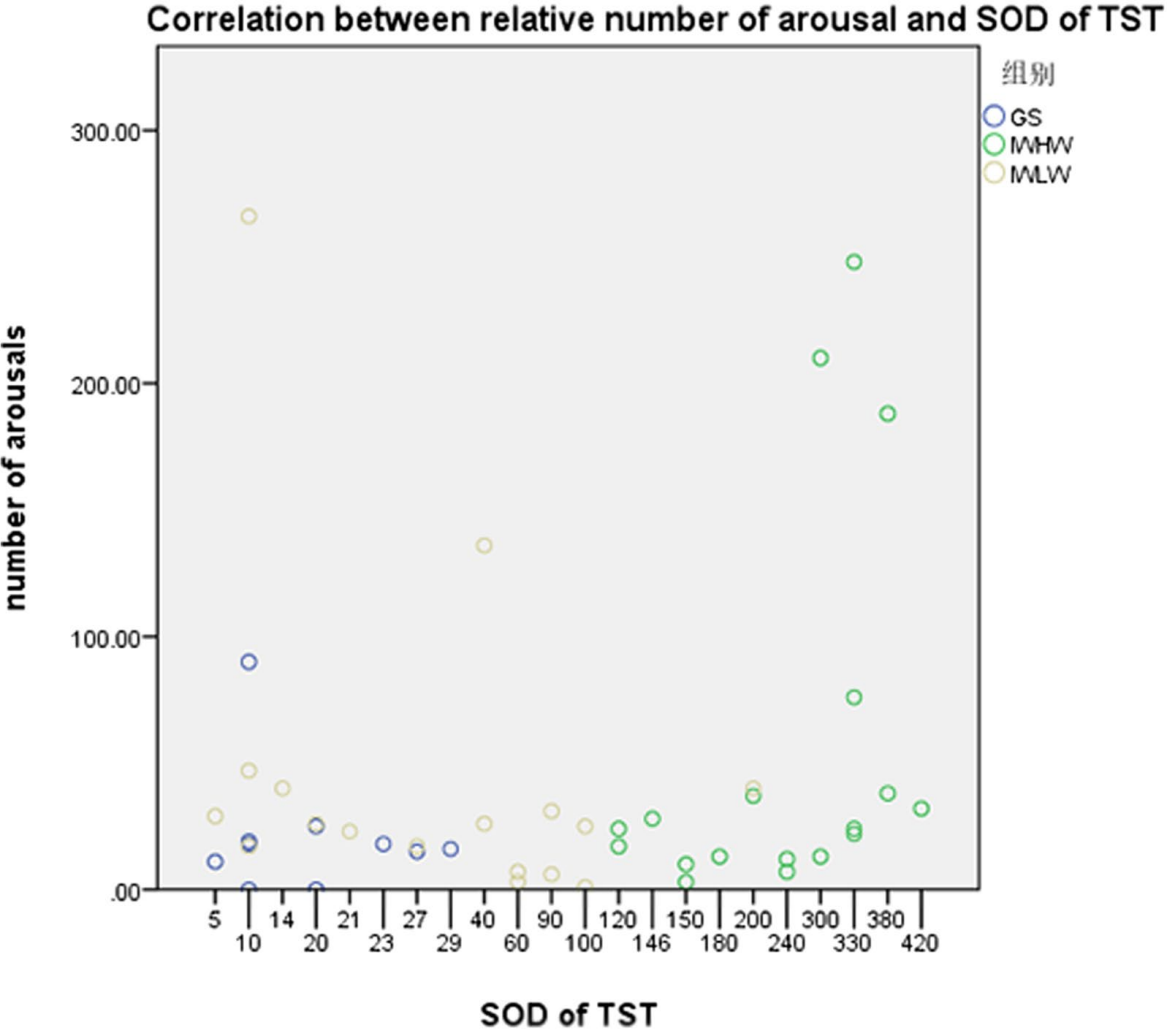
Correlation between the number of arousals and SOD of TST. SOD, Subjective–objective sleep discrepancy; TST: total sleep time; GS: good sleeper; IWLM: insomnias with a low mismatch; IWHM: insomnias with a high mismatch

Spearman’s correlation was performed on frontal beta and the SOD due to the non-normality of the data. The number of arousals was correlated with the SOD of TST (r = 0.532, *P* = 0.023) in the IWHW group (Table 7, Fig. 5).

**Table 7:** Correlation between the number of arousals and frontal beta and SOD of TST

Variables	Number of arousals	
	r	*P*
GS (n = 10)		
Absolute beta	− 0.049	0.894
Relative beta	− 0.482	0.159
SOD of TST	− 0.028	0.938
IWHM (n = 18)		
Absolute beta	0.271	0.277
Relative beta	0.253	0.311
SOD of TST	0.532	0.023
IWLM (n = 19)		
Absolute beta	0.395	0.094
Relative beta	0.167	0.495
SOD of TST	− 0.190	0.435

SOD: Subjective–objective sleep discrepancy

### Correlation between number of arousals and central beta, SOD of TST

Spearman’s correlation was performed on the SOD of TSTS due to the non-normality of the data. The number of arousals was correlated with the SOD of TST (r = 0.532, *P* = 0.023) in the IWHW group (Table 8).

**Table 8:** Correlation between the number of arousals and central beta and SOD of TST

Variables	Number of arousals	
	r	*P*
GS (n = 10)		
Absolute beta	0.055	0.880
Relative beta	− 0.085	0.815
SOD of TST	− 0.028	0.938
IWHM (n = 18)		
Absolute beta	0.413	0.089
Relative beta	− 0.030	0.906
SOD of TST	0.532	0.023
IWLM (n = 19)		
Absolute beta	0.173	0.479
Relative beta	− 0.155	0.527
SOD of TST	0.190	0.435

SOD: Subjective–objective sleep discrepancy

## Discussion

To the best of our knowledge, this is the first study that has investigated the absolute and relative spectral power of young adult patients (18–40 years old) with subtypes of subjective insomnia. Here, we categorized insomnia patients into IWLM and IWHM groups to maximize the difference in the SOD. Overall, compared to the GS group, patients with IWHM exhibited an increase in the absolute power and relative beta/delta ratio in the frontal region during sleep. Moreover, the SOD of TST was positively correlated with the absolute power and relative beta/delta ratio in the same frontal region. However, no significant difference was observed in the EEG power or beta/delta ratio in the central region and no significant difference in the above parameters was observed between the IWHM and IWLM groups.

Beta power is generally considered an indicator of cortical arousal. It has been shown that beta activity in PI patients is higher than that in GS [14–17], which suggests that patients with subjective insomnia may experience enhanced sensory processing during sleep. In fact, this phenomenon may render them highly responsive and sensitive to external sounds and in turn may also lead to the mistaken perception of their sleep as wakefulness [13]. Based on prior studies, sigma activity (sleep spindle) represents a marker of sleep stability, especially against noises [15]. Therefore, sigma activity may be able to distort the transmission of auditory information to the cortex during sleep [28]. A study from Spiegelhalder et al. [15] proposed the concept of simultaneous activation of wake-promoting and sleep-protecting neural activity patterns in PI. However, a meta-analysis showed that sigma increase during NREM sleeps in PI exhibited moderate and high heterogeneity in the dispersion of effect sizes [18]. Here, we found that both the IWHM and IWLM groups exhibited no increase in absolute and relative power of all frequency bands in the central and frontal regions. Our findings are similar to a previous EEG-based spectral investigation by Buysse et al. [29] that failed to find significant differences in the frequency band activity between insomnia types and GS during NREM sleep. Nevertheless, unlike other previous work, we failed to observe lower delta NREM EEG activity, or greater alpha, theta, sigma, beta NREM EEG activity in patients with insomnia. This discrepancy was unclear but could be influenced by the difference in the age, frequency band definitions and diagnostic criteria of IWHM and IWLW patients in the two studies. To our knowledge, there has been little research on the relationship between age and power spectra, leading to dissimilar results. For example, Krystal et al. [19] reported that older age (40–80 years-old) was associated with significantly lower sigma (12.5–16 Hz) relative power during NREM in insomnia patients. Svetnik et al. [23] demonstrated that the power of the delta, theta and sigma bands significantly decreased with age whereas the slope in the alpha, beta and gamma bands did not. Therefore, age may be a potential influencing factor.

Insomnia is associated with poorer cognitive performance both generally and across multiple specific cognitive domains, especially in terms of a decline of working memory and executive ability [30, 31]. A longer course of insomnia generally leads to a poorer cognitive impairment, which manifests as slower EEG frequency, a higher proportion of alpha and beta band power, and a lower proportion of theta and delta band power. It will be more conducive if the relationship between age and power spectrum could be studied in combination with the course of disease. To our knowledge, PSA studies categorizing insomnia into subtypes are limited. Some studies determined if the PSG was normal as a basis for judging subjective and objective insomnia [19], which may have led to the inclusion of patients with different subtypes of insomnia. Other studies have also explored SOD of SOL. In our research, insomnia patients were further categorized into two subgroups based on their SOD of TST. IWLM individuals exhibited a SOD < 60 min in TST whereas IWHM individuals exhibited a SOD > 120 min in TST. In our study, the PSG of patients with IWHM and IWLW was normal and the PSQI was higher than GS participants. This meant that all patients were of subjective insomnia, but that the degree of SOD was different. SOD of IWHW insomnia patients was greater than 120 min in TST, while that of IWLW insomnia patients was less than 120 min. In addition, patients with subjective insomnia met persistent PI criteria and had a normal single night PSG in Krystal’s paper, which did not clearly define SOD.

Recently, the ratio of high-frequency to low-frequency EEG power has been recognized as a novel indicator of cortical arousal. Furthermore, individuals with a higher ratio of this sort may have more sleeping difficulties. Meric et al. [32] found that PsyI patients exhibited an increased beta/delta ratio in the temporal lobe during the sleep onset period (SOP). Some studies have also reported that delta EEG activity is decreased in PI patients in the temporal and central brain regions during the SOP [33, 34]. Thus, such an activity index (beta/delta ratio) may be a more appropriate indicator of cortical arousal in insomnia patients [17, 32, 35]. In the current study, IWHM patients showed increased absolute power and relative beta/delta ratio in the frontal region compared with the GS group, suggesting hyperarousal in the frontal portion of the brain.

SOD in insomnia has been shown to arise due to several possible mechanisms, which mainly focus on sensory perception, emotion and cognition [9]. Various characteristics of insomnia patients support these concepts, such as: (1) insomniacs will judge PSG measured sleep as wakefulness; (2) insomniacs have anxiety and selective attention toward sleep-related threats. The possibility that anxiety serves to trigger the misperception of sleep is drawn from the robust finding in time perception literature in that time is perceived as longer when the number of units of information processed per unit of time increases. Other characteristics of insomnia patients include: (3) patients may simply be poor estimators of time; and (4) insomniacs' assessment of sleep quality is influenced by a memory bias that is influenced by current symptoms and emotions, a confirmation bias/belief bias or a recall bias linked to intensity. In many other papers, central regions, mainly involving in sensory perception, are considered good representations of the whole brain activity (from EEG) and have been widely used in PSA. Frontal lobes are also related to emotion, cognition, and behavioral management, which is connected with the mechanism of SOD. Therefore, it is necessary to assess frontal regions. Unfortunately, cortical activation at sites other than central areas, such as frontal regions, has been poorly explored. In our study, a higher beta/delta ratio was only observed in frontal regions in the IWHW group when comparison to GS group. This result seems to suggest that high cortical arousal occurs in the frontal lobe and not just in the central region.

We further showed that the SOD of TST was associated with the absolute and relative NREM beta/delta ratio (r = 0.363) and relative beta power (r = 0.313) in the frontal area. All in all, these results indicate that a higher the beta/delta ratio and beta power during NREM sleep may be an underestimation of TST. Our results are similar to the findings by Perlis et al. [14] that showed a moderate correlation between the SOD of TST and NREM beta activity (14–35 Hz) (r =  − 0.46). The underestimation of TST may be explained by the insertion of high frequency EEG into low frequency EEG, which has been shown to enhance the information processing ability and to degrade sleep quality [36].

To the best of our knowledge, few studies have reported the correlation between the SOD of TST and the number of arousals. Results from our study showed that the number of arousals was correlated with the SOD of TST in the IWHW group, suggesting that frequent awakenings that lead to sleep fragmentation may in turn lead to poor perception of insomnia. This is similar to the previous study by Choi et al. [37] that showed that sleep perception was negatively related to the PSG arousal index.

There are various limitations to our study that must be noted. First, only one PSG recording was performed in each participant and thus, the results might be biased by the “first night” effect. Secondly, insomnia patients were categorized into the IWHM and IWLM groups based only on TST. The percentage of SWS should also be considered in future investigations. Lastly, the sample size in this work was relatively small. Future studies with larger sample sizes are needed to further elucidate the neurophysiological mechanisms about the SOD.

## Conclusion

In conclusion, the present study suggests that IWHM insomnia in young adults is associated with increased absolute power and relative beta/delta ratio in the frontal brain region. However, there was no significant difference observed between the IWHM and IWLM groups. Furthermore, the SOD of TST was associated with the frontal NREM beta/delta ratio. This indicate that the increased beta/delta ratio is a characteristic of sleep misperception. Finally, it is necessary to look at both frontal and central regions when investigating the mechanism of insomnia.

## Data Availability

The datasets used and/or analysed during the current study are available from the corresponding author on request.
